# P-loop Conformation Governed Crizotinib Resistance in G2032R-Mutated ROS1 Tyrosine Kinase: Clues from Free Energy Landscape

**DOI:** 10.1371/journal.pcbi.1003729

**Published:** 2014-07-17

**Authors:** Huiyong Sun, Youyong Li, Sheng Tian, Junmei Wang, Tingjun Hou

**Affiliations:** 1 Institute of Functional Nano & Soft Materials (FUNSOM) and Collaborative Innovation Center of Suzhou Nano Science and Technology, Soochow University, Suzhou, Jiangsu, China; 2 College of Pharmaceutical Sciences, Zhejiang University, Hangzhou, Zhejiang, China; 3 Department of Biochemistry, University of Texas Southwestern Medical Center, Dallas, Texas, United States of America; University of Houston, United States of America

## Abstract

Tyrosine kinases are regarded as excellent targets for chemical drug therapy of carcinomas. However, under strong purifying selection, drug resistance usually occurs in the cancer cells within a short term. Many cases of drug resistance have been found to be associated with secondary mutations in drug target, which lead to the attenuated drug-target interactions. For example, recently, an acquired secondary mutation, G2032R, has been detected in the drug target, ROS1 tyrosine kinase, from a crizotinib-resistant patient, who responded poorly to crizotinib within a very short therapeutic term. It was supposed that the mutation was located at the solvent front and might hinder the drug binding. However, a different fact could be uncovered by the simulations reported in this study. Here, free energy surfaces were characterized by the drug-target distance and the phosphate-binding loop (P-loop) conformational change of the crizotinib-ROS1 complex through advanced molecular dynamics techniques, and it was revealed that the more rigid P-loop region in the G2032R-mutated ROS1 was primarily responsible for the crizotinib resistance, which on one hand, impaired the binding of crizotinib directly, and on the other hand, shortened the residence time induced by the flattened free energy surface. Therefore, both of the binding affinity and the drug residence time should be emphasized in rational drug design to overcome the kinase resistance.

## Author Summary

Cancers can eventually confer drug resistance to the continued medication. In most cases, mutations occurred in a drug target can attenuate the binding affinity of the drugs. Here, we studied the drug resistance mechanisms of the mutations G2032R in the ROS1 tyrosine kinase in fusion-type NSCLC. It is well known that the phosphate-binding loop (P-loop) plays a vital role in the binding of competitive inhibitors in tyrosine kinases, and numerous mutations have been found occurred around the P-loop, which may affect the binding/unbinding process of a drug. Free energy surfaces were constructed to characterize the impact of the mutation to the binding/unbinding process of a well-known NSCLC drug, crizotinib. Two advanced free energy calculation methods, namely funnel based well-tempered metadynamics and umbrella sampling based absolute binding free energy calculation achieved consistent results with the experimental data, suggesting that the rigid P-loop of the mutated target was mainly responsible for the crizotinib resistance to ROS1 tyrosine kinase.

## Introduction

The past decade has witnessed the great benefit of the personalized drug therapy in the treatment of non-small-cell lung cancers (NSCLC) [1]–[3], which was designed to target different drug targets, such as KRAS [4], EGFR [5], EML4-ALK [6], the newly found CD74-ROS1 [7], [8], etc. Crizotinib, the latest launched NSCLC drug, was originally designed to competitively inhibit the activity of c-MET [9], whereas has been approved by U.S. Food and Drug Administration (FDA) for the treatment of advanced NSCLC with anaplastic lymphoma kinase (ALK) rearrangements in 2011. And recently, it has also been found with great clinical benefit in the treatment of advanced NSCLC patients with fusion-type CD74-ROS1 tyrosine kinase with the response rate of 57% and a disease control rate at 8 weeks of 79% [10], [11]. Therefore, crizotinib may be the most successful chemical drug for the personalized therapy in NSCLC.

Unfortunately, under strong purifying selection, cancer cells can eventually confer resistance to the therapeutic drugs, and they may survive by means of activating other signaling pathways [12]–[16], regulating the expression level of the associated genes or gene products [17]–[19], or more directly, hindering the drugs binding [20], [21], enhancing the substrates binding [22], or re-activating the target [23] with acquired secondary mutations in the drug target. Therefore, it is no surprise that ROS1 was trapped in the crizotinib resistance as well, with very short term of the crizotinib therapy as reported by Awad and colleagues [24]. They had found a *de novo* secondary mutation G2032R in CD74-ROS1, and this mutation conferred serious resistance to crizotinib. It was supposed that the mutation was located at the solvent front, and might hinder the drug binding. However, it might not be true when one has a view on the crystal structure, where a large binding pocket can be found in the drug-target complex, and actually, a sole mutation may hardly hinder the drug binding as we showed below (the drug could smoothly unbind or rebind to the mutated ROS1 tyrosine kinase). Alternatively, by using advanced molecular dynamics (MD) methodologies (funnel based well-tempered metadynamics and Woo and Roux's absolute binding free energy calculation scheme), we constructed the free energy surfaces (FESs) along the drug-target distance and the phosphate-binding loop (P-loop) conformational change which is responsible for the binding of competitive inhibitors to tyrosine kinases, and the FESs unrevealed the drug resistance mechanism in detail: the more rigid P-loop region in the G2032R mutant was the main reason for the crizotinib resistance, which on one hand, impairs the binding of crizotinib directly, and on the other hand, shortens the residence time as well. Therefore, considering the importance of the role of kinases in the therapy of carcinomas, we suggests that, besides emphasizing the binding affinity, the residence time should be considered to design potent leads to overcome resistance as well.

## Results

### Structural Change of Bound-State and Unbound-State ROS1 Tyrosine Kinases in Conventional Molecular Dynamics Simulations

**Figure 1:**
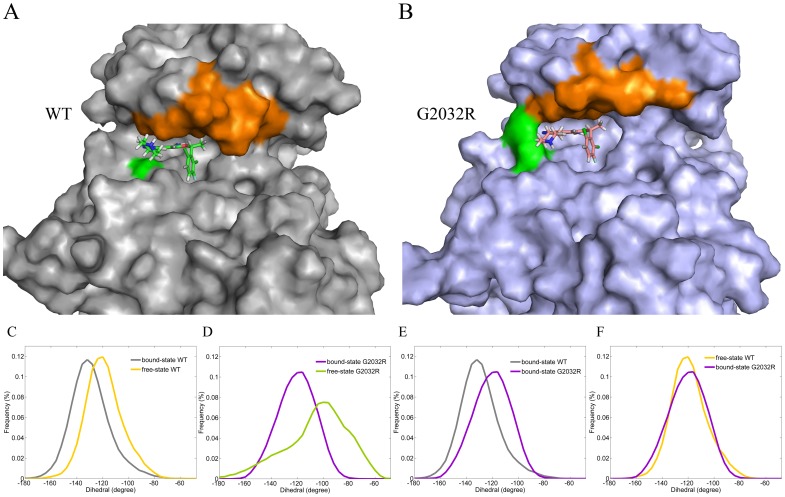
The most populated bound-state conformations (averaged structures) and dihedral angle distributions of the P-loop region in WT-ROS1 (gray, panel A) and G2032R-ROS1 (purple, panel B). The dihedral angle was calculated by C_α_ of the residues 20, 22, 25 and 150 (the index of the residues were renumbered from 1 to 285) in panels C–F. The mutated site G2032R, P-loop region, and crizotinib in WT-ROS1 and G2032R-ROS1 are shown in green surface, orange surface, green stick, and pink stick models, respectively, in panels A and B. The dihedral angle distributions are colored in grey, orange, purple, and green in bound-state WT-ROS1, free-state WT-ROS1, bound-state G2032R-ROS1, and free-state G2032R-ROS1, respectively.

As shown in Figure S1, all the systems (bound-state WT-ROS1, free-state WT-ROS1, bound-state G2032R-ROS1 and free-state G2032R-ROS1) reached equilibrium after 5 ns simulation, with the RMSDs (A and B) less than 3 Å and RMSFs (C and D) less than 2 Å in most regions. Therefore, the equilibrated trajectories (5∼30 ns) were suitable for the conformational analysis, and the following metadynamics and umbrella sampling simulations. Although no free-state ROS1 tyrosine kinase was crystallized, we could construct a free-state ROS1 by removing the co-crystallized ligand in the crystal structure instead. It is well-known that the binding site of a target could be induced into a suitable conformation when binding with a ligand, the so called induced fit phenomenon, and we detected the conformational difference between the bound-state and unbound-state ROS1 (WT-ROS1 and G2032R-ROS1) in the P-loop region as well. As shown in Figure 1C, the most populated dihedral angle of the P-loop in the bound-state WT-ROS1 is ∼20° smaller than that in the free WT-ROS1, suggesting that a more closed state of the P-loop was prevalent in the bound-state protein, which is consistent with the induced fit theory. The same phenomenon has been observed in G2032R-ROS1 in Figure 1D, where a very different distribution of the dihedral angle of the P-loop region was detected between the bound-state (purple) and free-state (green) G2032R-ROS1, with the middle value 20° larger and more widely distributed in the unbound-state G2032R-ROS1. That is to say, the mutation makes the P-loop region of the free-state G2032R-ROS1 more flexible, and a more opened structure of the P-loop region is dominant in the free G2032R-ROS1.

**Figure 2:**
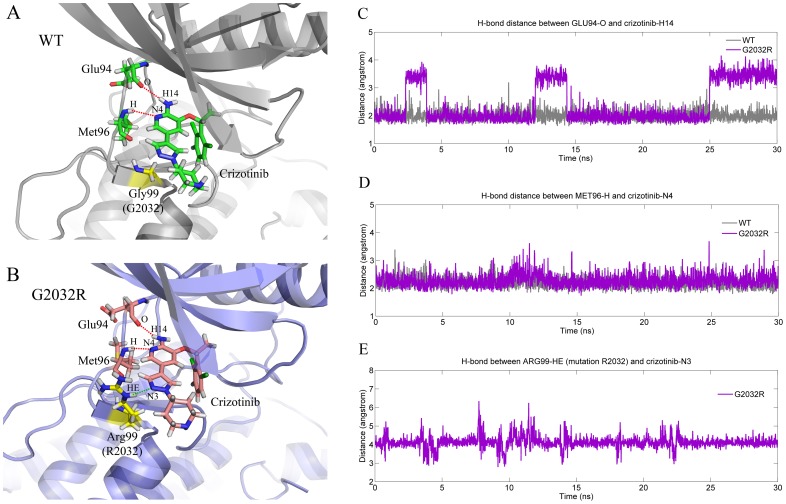
H-bond interactions between crizotinib and ROS1 tyrosine kinase. Two and three stable H-bonds were found in WT-ROS1 (A) and G2032R-ROS1 (B), respectively. The mutated residue was colored in yellow, and it can be found that a new hydrogen bond was formed between R2032 and crizotinib in G2032R-ROS1 (green dot line). The time evolutions of the H-bond distance changes were plotted in panels C, D, and E, where the H-bond in WT-ROS1 and G2032R-ROS1 were colored in gray and purple, respectively.

A comparison of the bound-state WT-ROS1 (gray) and G2032R-ROS1 (purple) shows that the P-loop region in G2032R-ROS1 is indeed more opened than that in WT-ROS1 (Figure 1E), and the difference of the dihedral angle is ∼20°, which is close to the angular difference between the bound-state and free WT-ROS1 (Figure 1C). Therefore, a comparison was carried out between the dihedral angle distributions of the unbound-state WT-ROS1 and bound-state G2032R-ROS1. As shown in Figure 1F, interestingly, similar distributions of the dihedral angles were found for the unbound-state WT-ROS1 (orange) and bound-state G2032R-ROS1 (purple), indicating the P-loop regions in the unbound-state WT-ROS1 and bound-state G2032R-ROS1 adopted similar conformations. The averaged structures of the bound-state WT-ROS1 (Figure 1A) and G2032R-ROS1 (Figure 1B) show that the P-loop region (orange in Figure 1A and 1B) in G2032R-ROS1 is indeed upper-moved compared with that in WT-ROS1, and this phenomenon could be attributed to the mutation G2032R directly, which formed a scaffold-like structure (green region in Figure 1B) and supported the P-loop region in G2032R-ROS1. On the contrary, the no-side-chain amino acid glycine in WT-ROS1 (green in Figure 1A) cannot affect the conformation of the P-loop region anymore. Therefore, it could be found (Figure 1A and 1B) that the binding pocket in G2032R-ROS1 was more opened than that in WT-ROS1, and still, the pyridine ring of crizotinib (pink stick model) near the mutated site (green) in G2032R-ROS1 was located more outside of the binding pocket compared with the fragment (green stick model) in WT-ROS1. Awad has supposed that the solvent front mutation G2032R may hinder the drug binding to the mutated ROS1 [24], which seems possible when a small amino acid was replaced by a larger one. However, the fact that the drug has similar binding pose to that of WT-ROS1 (Figure 2A), and most part of the drug is still located in the active pocket of G2032R-ROS1 (Figure 2B). Moreover, no hydrogen bonds were lost (Figure 2C and 2D) or even a new hydrogen bond was formed between the mutated residue R2032 and crizotinib (Figure 2E, green dot line), indicating that the mutation cannot directly hinder the drug binding. Alternatively, it seems that the up-moved P-loop region in G2032R-ROS1 could attribute to the crizotinib resistance by attenuating the interactions between the drug and the enlarged binding pocket, which has also been observed in the C1156Y induced crizotinib resistance in the ALK tyrosine kinase [25]. Therefore, by using advanced free energy calculation approaches, we discussed the drug resistance mechanism in detail in the next two sections.

### Unbinding Pathways of Crizotinib from ROS1 Tyrosine Kinase Characterized by Two-Dimensional Free Energy Surfaces

Drug resistances are usually associated with the attenuation in drug binding to its target, which can be mechanically detailed by free energy calculations. Although two-end-state calculations are effective in determining the change of the total binding free energies between the mutated and the wild-type drug-target complexes [26]–[29], it is indeed powerless in describing a physically associated pathway upon how a drug binds to or unbinds from its target, which may be more helpful in understanding the binding or unbinding process of a drug. Alternatively, process-based methods can easily solve the problem by calculating a one-dimensional (1D) free energy profile or a two-dimensional (2D) free energy surface along given reaction coordinates (RCs).

**Figure 3:**
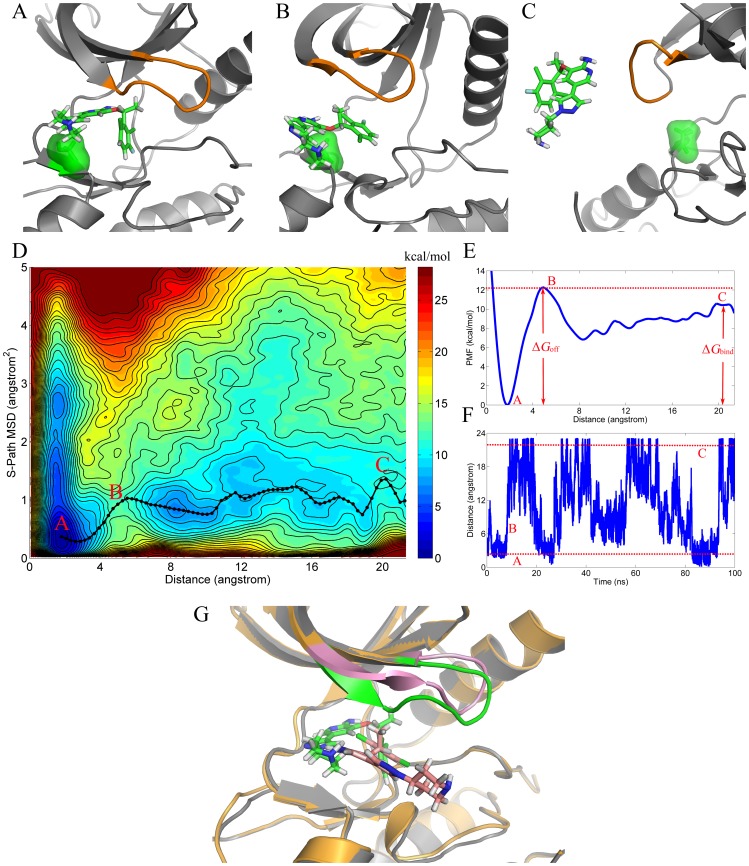
Free energy surface (FES, kcal/mol) of crizotinib separated from WT-ROS1 (D). X-axis and Y-axis denote the separation distance between the CoMs (center of mass) of crizotinib and the binding site of ROS1 tyrosine kinase (heavy atoms within 5 Å of the drug) and the similar path mean-square-deviation (MSD) of the P-loop region (a large MSD represents the unbinding conformation), respectively. The 2D FES was mapped into a 1D free energy profile (Panel E) using the minimum-free-energy pathway as shown in black-dot line in Panel D. Panels A, B, and C are structural description of the corresponding red point shown in Panel D, where the P-loop region was colored in orange, and the pre-mutated site (G2032) and crizotinib are shown in green surface model and green stick model, respectively. Time evolution of the crizotinib unbinding and rebinding to WT-ROS1 is illustrated in Panel F. The structures of the corresponding red points (points A and B) in Panel D are overlapped (Panel G) to highlight the conformational change of the P-loop region (green in point A and pink in point B in Panel D).

Here, funnel based well-tempered metadynamics was employed to construct the free energy surfaces. In the spirit of metadynamics, repulsive Gaussian potentials were periodically added to the selected reaction coordinates, and therefore, the biased molecule could unbind and rebind to the active pocket repeatedly. As illustrated in Figure 3F, the drug was periodically binding (bound-state, red dot line A) to and unbinding (overcome-barrel state, B, and free-moving state, red dot line C) from the active pocket, which represents the convergence of the binding-unbinding process, and the added potentials could be used for the FES construction. As shown in Figure 3D, the 2D free energy surface was plotted with the CoMs distance (the centers of masses between crizotinib and the active pocket of ROS1) as X-axis and the P-loop conformational change (similar path mean-square-deviation of P-loop) as Y-axis. In X-axis, a position near 0 Å denotes the drug bound in the active pocket, while the drug could move freely in bulk at the position of 20 Å. Similar path MSD was employed to detect the conformational change of P-loop when it binds or unbinds the drug. A lower value of the S-path MSD means that the conformation of P-loop is very similar to that of the bound-state P-loop, while a higher value represents the free-state P-loop or much-opened P-loop. In the FES, the area was colored from blue to red where has the lowest and highest ensemble energy, e.g. ligand in the binding site and bulk. The minimum-free-energy pathway (black dot line in Figure 3D) was constructed by connecting the bins with the minimum free energies along the CoM distance (X-axis). Interestingly, an induced fit behavior was observed during the drug binding and unbinding processes from the active pocket, where the P-loop closed when crizotinib binding to the active pocket (lower S-path MSD), and opened when crizotinib freely moved in bulk (higher S-path MSD). More detailed structural descriptions have been illustrated in Figures 3A, 3B, and 3C, which correspond to the stable bound-state (red point A in Figure 3D), overcome-barrier state (red point B in Figure 3D), and free-moving state (red point C in Figure 3D), respectively. Figure 3E represents the longitudinal section of the 2D FES, and a high barrier was found located at 4∼8 Å of the RC (position B), indicating that high energy was needed to overcome the barrier when crizotinib got into or out of the binding site. The overlapped structures of the bound-state (gray cartoon model) and overcome-barrier state (orange cartoon model) WT-ROS1 complexes uncovered the high barrier mechanism (the induced fit phenomenon). As shown in Figure 3G, a large conformational change of the P-loop region was observed in the overcome-barrier state WT-ROS1 (pink cartoon region), which was markedly up-moved compared with that of the bound-state WT-ROS1 (green cartoon region), and this needed much energy to cancel the conformational energy loss of P-loop.

**Figure 4:**
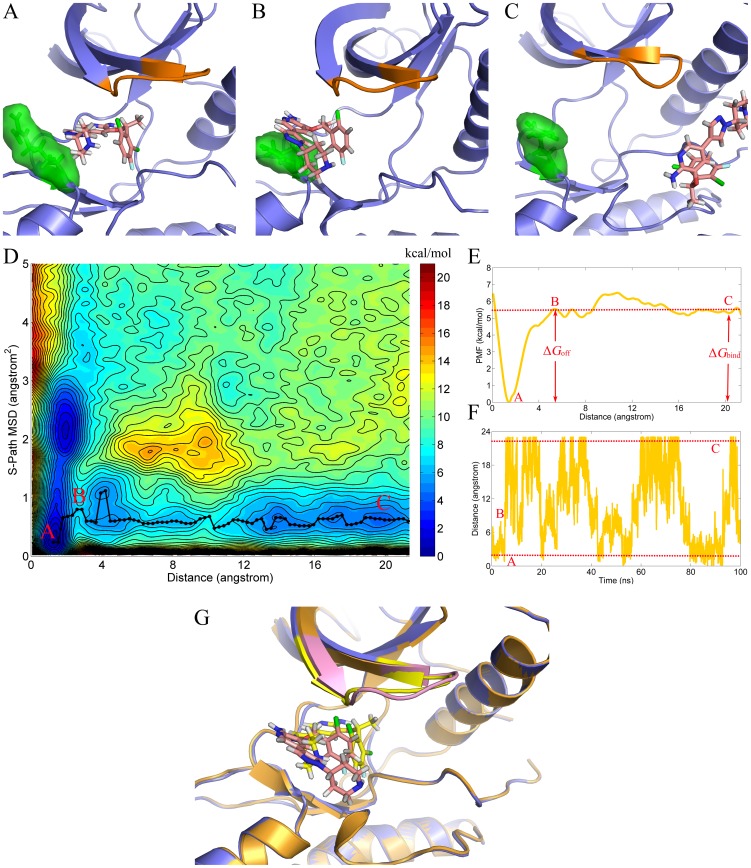
Free energy surface of crizotinib separated from G2032R-ROS1 (D). 1D free energy profile (Panel E) was constructed from the minimum-free-energy points as shown in black-dot line in Panel D. Panels A, B, and C are structural descriptions of the corresponding red points in Panel D, where the P-loop region was colored in orange, and the mutated site (R2032) and crizotinib are shown in green surface model and pink stick model, respectively. Time evolution of the crizotinib unbinding and rebinding to G2032R-ROS1 is illustrated in Panel F. The structures of the corresponding red points (point A and B) in Panel D were superimposed (Panel G) to highlight the conformational similarity of P-loop (yellow in point A and pink in point B in Panel D).

Similar behavior of crizotinib was found in the G2032R mutated ROS1 tyrosine kinase. As shown in Figure 4F, the drug periodically bound into and unbound from the active pocket as well, and a same process was observed of the drug unbinding from the target, where the pyridine ring was first getting out of the binding site and followed by the halogenated benzene fragment (Figure 4A, 4B, and 4C). However, the free energy surface was different from that of WT-ROS1 to a certain extent. At first, there was no energy barrier located at 4∼8 Å of the CoM-distance based RC (X-axis) as shown in Figure 4E (point B). Second, the most favorable unbinding pathway (minimum-free-energy pathway) showed a much lower S-path MSD value (∼0.5 Å^2^) in G2032R-ROS1compared with that in WT-ROS1 (>1 Å^2^). Structural observation showed that, unlike WT-ROS1, there was no conformational change of the P-loop region when the drug unbinding form the active pocket as illustrated in Figure 4G, where the bound-state and overcome-barrier state G2032R-ROS1 complexes were shown in purple (yellow in P-loop) and orange (pink in P-loop) cartoon models, respectively. Therefore, no energy was needed to cancel the energy loss associated with the conformational change of P-loop. Besides, as discussed above, the conformation of the P-loop region in the bound-state G2032R-ROS1 is very similar to the free-state P-loop in WT-ROS1, indicating that no much conformational change was needed to bind or unbind the drug in G2032R-ROS1. As a result, slight change of the S-path MSD was observed in the bound-state (point A in Figure 4D) and unbound-state (point C in Figure 4D) G2032R-ROS1.

### Crizotinib Resistance Mechanism Validated and Uncovered in Detail by Consistence of One-Dimensional Absolute Binding Free Energy Profiles

Although metadynamics simulations have been widely used in FES construction, it will be hard to get a convergent result when missing any associated reaction degree of freedom. Therefore, by using the absolute binding free energy calculation based on the umbrella sampling (US) simulations, we validated the consistence of our results, and obtained a more stable prediction of the binding free energy based on the minimum-free-energy pathway derived from the metadynamics simulations.

**Figure 5:**
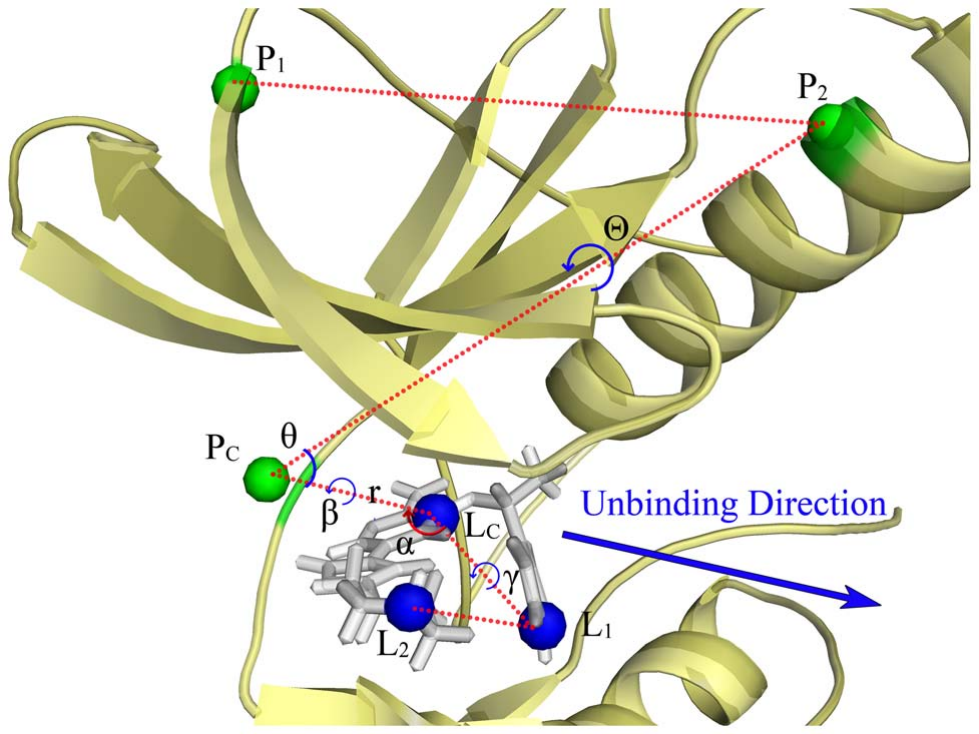
Rotational and translational restrains on crizotinib. The defined reference points are colored in green (P_1_, P_2_, and P_C_) in the ROS1 tyrosine kinase (yellow) and blue (L_1_, L_2_, and L_C_) in crizotinib (white), respectively. P_1_ and P_2_ points are C_α_ of Leu12 and Lys58, respectively, and P_C_ point is a fictitious atom sited 5 Å away from L_C_ along X-axis. L_1_, L_2_, and L_C_ are the atoms of C19, N1, and C12, respectively, in crizotinib. The rotational restrains are defined as α(L_1_, L_C_, P_C_), β(L_1_, L_C_, P_C_, P_2_), and γ(L_2_, L_1_, L_C_, P_C_), and the translational restrains are used θ(L_C_, P_C_, P_2_), Θ(L_C_, P_C_, P_2_, P_1_), and r (along the vetor of 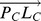, also defined as X-axis, which is the unbinding direction of crizotinib as shown in blue arrow).

**Figure 6:**
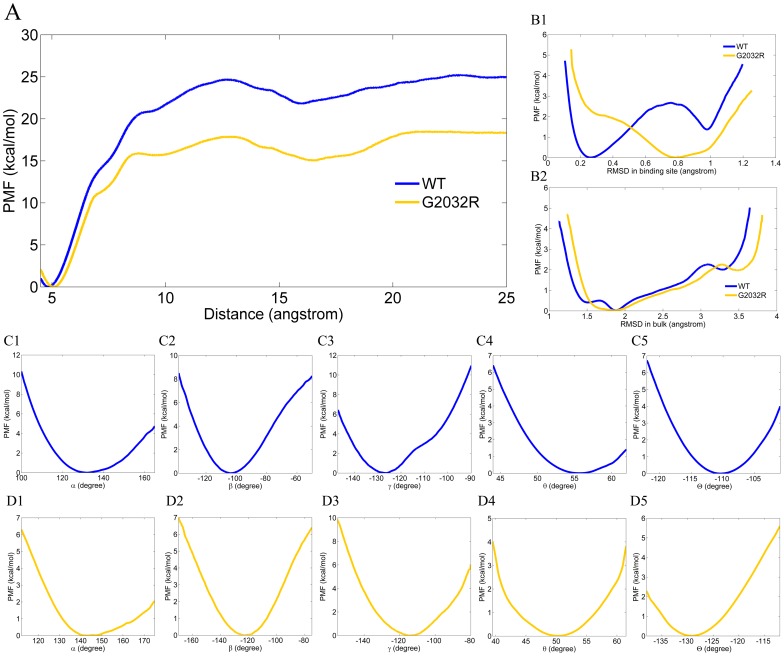
1D free energy profiles of crizotinib separated from WT-ROS1 (A, blue) and G2032R-ROS1 (A, orange). The separation simulations were under the existence of conformational (RMSD, B1 in binding site, and B2 in bulk), rotational (C1 and D1 for angle α, C2 and D2 for dihedral angle β, C3 and D3 for dihedral angle γ), and translational (C4 and D4 for angle θ, C5 and D5 for dihedral angle Θ) restrains. The PMFs are colored in blue and orange in WT-ROS1 and G2032R-ROS1, respectively.

Woo and Roux's scheme [30] was employed to calculate the absolute binding free energy. The starting structure of each window (a total of 40 windows) of the separation US simulation was derived from the metadynamics simulations, which have constructed the most favorable unbinding pathway of crizotinib [31]. As shown in Figure 5, six points were selected to control the rotational and translational degree of freedoms of the crizotinib unbinding process, where the point P_C_ was substituted by a fictitious atom placed at 5 Å away from L_C_ along X-axis. Therefore, it can be found in Figure 6A that the minimum energy points of WT-ROS1 and G2032R-ROS1 are both located at 5 Å of the RC. In the restrained US simulations, the conformation-dependent PMFs of crizotinib were first calculated in the binding site (Figure 6B1) and bulk (Figure 6B2). As shown in Figure 6B1, the shape of the PMF line in G2032R-ROS1 (orange) has a much broader local minima region compared with that in WT-ROS1, indicating that a more loose binding conformation was employed by G2032R-ROS1. This inference has been validated by our analysis above that a more opened P-loop was dominated in the bound-state G2032R-ROS1, which leads to the loose-binding of crizotinib. Compared with the bound-state RMSD based PMFs, a much broader RMSD change of crizotinib was found in bulk (Figure 6B2). Although different starting conformations of crizotinib were employed for the conformation-restrained simulations in WT-ROS1 and G2032R-ROS1, a very similar shape of the RMSD based PMFs was observed for WT-ROS1 and G2032R-ROS1 (individualized bound-state crizotinib was used as the reference conformation for the restrained US simulation), which means that consistent binding conformations of crizotinib were used inWT-ROS1 and G2032R-ROS1. Due to the existence of the conformational restrains in crizotinib, no significant difference has been observed in the angle based PMFs (α, β, γ, θ, and Θ) between WT-ROS1 (Figure 6C1–6C5) and G2032R-ROS1 (Figure 6D1–6D5). However, large difference has been found between the separation PMFs of WT-ROS1 (blue) and G2032R-ROS1 (orange). As shown in Figure 6A, the separation PMF in G2032R-ROS1 was much lower than that in WT-ROS1, suggesting that serious crizotinib resistance could be induced by the mutation G2032R in the ROS1 tyrosine kinase.

The energetic component contributions have been summarized in Table 1, where the conformational restrains (binding site, 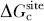, and bulk, 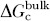), rotational restrains (binding site, 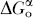, 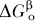, and 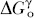, and bulk, 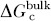), and translational restrains (binding site, 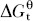 and 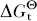) associated energies were calculated by the direct integration of the Boltzmann factor based on the PMFs (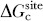, 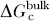, 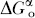, 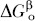, 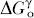, 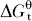, and 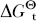) or numerical integration (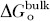). The separation PMFs (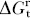) were obtained by finding the energy difference between the bound-state and unbound-state ensembles. As listed in Table 1, all the energetic components contributed slightly to the difference of the total energies except the separation PMF. Therefore, the separation PMF difference should be the main contributor for the crizotinib resistance, which is consistent with the analysis shown above that the opened structure of P-loop in G2032R-ROS1 leads to more loose binding of the drug. Although the predicted binding free energies given by the two methodologies (US based binding free energy, Δ*G*_bind-US_, and metadynamics based binding free energy using minimized pathway, Δ*G*_bind-Meta_) are a bit different, the binding free energies both markedly decreased in G2032R-ROS1, which is consistent with the experimental data of IC_50_.

**Table 1:** Free energy decomposition of the absolute binding free energy (kcal/mol).

Free energy decomposition	WT-ROS1	G2032R-ROS1	ΔΔ*G*
**Woo and Roux's scheme based on umbrella sampling**			
Conformational restrain in binding site, 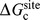	0.073	0.323	0.250
Conformational restrain in bulk, 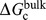	1.639	1.772	0.133
Orientational restrain in angle α, 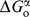	0.410	0.617	0.207
Orientational restrain in dihedral angle β, 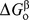	0.892	0.520	−0.372
Orientational restrain in dihedral angle γ, 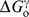	0.210	0.389	0.179
Orientational restrain in bulk, 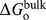	7.135	7.230	0.095
Translational restrain in angle θ, 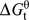	0.355	0.214	−0.141
Translational restrain in dihedral angle Θ, 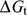 ^Θ^	0.162	0.165	0.003
Translational PMF along CoMs, 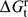	−24.96	−18.32	−6.64
S*	9.834	8.950	-
I*	1.98e+017	4.44e+012	-
*K*_bind_	3.85e+013	6.64e+008	-
Δ*G*_bind-US_	−14.70±0.58^a^	−7.95±0.27	−6.75
**Funnel based well-tempered metadynamics**			
Δ*G*_off_	12.10	5.53	6.57
Δ*G*_bind-Meta_	−10.14±0.60^b^	−5.91±0.30	−4.23
**Experimental data based on IC_50_/K_d_** c			
IC_50_ [24] (nM)	2.1	570	-
Δ*G*_exp-Kd_ [105]	−13.35	-	-

a the deviations were estimated based on the last 4 ns US simulation of each window.

b the deviations were estimated based on the minimized pathway of metadynamics from 15 to 22 Å.

c the experimental binding free energy was estimated by Δ*G*_exp_ = −*RT*ln*K*_Kd_ at 310 K.

## Discussion

By using advanced molecular dynamics techniques, namely funnel based well-tempered metadynamics and umbrella sampling based absolute binding free energy calculation approaches, we investigated the drug resistance mechanism of G2032R in ROS1 tyrosine kinase. A more rigid conformation of the P-loop was detected in G2032R-mutated ROS1 tyrosine kinase, which was much opened even in the bound-state and had a similar conformation as that of the free-state wild-type ROS1 tyrosine kinase. The conformational analysis showed that the scaffold-like side chain of the mutation R2032 was responsible for the markedly opened structure of the P-loop in G2032R-ROS1, which supported the P-loop and hindered its closure during the drug binding. Therefore, the P-loop was hard to be induced during the whole binding/unbinding process of crizotinib, and thus, an attenuated binding state was dominated between crizotinib and binding pocket of G2032R-mutated ROS1 tyrosine kinase. In addition, we have analyzed the energetic contribution to crizotinib on residue level as well, which showed that the residue Leu18 (located just in the P-loop region) contributed the most to the attenuated binding of crizotinib to G2032R-ROS1 as shown in Figure S3, therefore well supporting the issue that the P-loop conformation governs crizotinib resistance in G2032R mutated ROS1 tyrosine kinase.

It has been discussed above that the up-moved P-loop has directly attenuated the binding of crizotinib to G2032R-ROS1, which corresponds to a substantial loss of the total binding free energy compared with WT-ROS1 (ΔΔ*G* of Δ*G*_bind-Meta_ and Δ*G*_bind-US_). Nevertheless, another reason may still contribute to the drug resistance, namely, the shortened residence time of crizotinib in G2032R-ROS1. As the notion describes, a larger activation free energy of dissociation, Δ*G*_off_, corresponds to a longer residence time: 
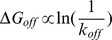
, where 1/*k*_off_ has been defined as the residence time (*t* = 1/*k*_off_). Therefore, a lead may be more promising to be a drug if it has a longer residence time in the organisms [32]–[35]. It can be found in Figure 3E that a large barrier was located at 4∼8 Å of the RC in WT-ROS1, which, although, might hinder the drug getting into the active pocket, it could indeed significantly increase the residence time with the Δ*G*_off_ of ∼12 kcal/mol, which is much larger than that in G2032R-ROS1 (Δ*G*_off_ = ∼5.5 kcal/mol, as shown in Table 1).

Taken together, it could be summarized that the up-moved P-loop region, which was supported by the scaffold-like conformation of the large side chain of R2032, has contributed mainly to the crizotinib resistance in G2032R-ROS1, where it, on one hand, decreased the binding affinity of the drug by loosed-binding-state because of the enlarged binding pocket, and on the other hand, shortened the residence time by flattening the free energy surface. The calculated binding free energy was reasonably consistent with the experimental data, suggesting that, besides binding affinity, residence time should be considered as well for rational drug design to overcome drug resistance.

## Materials and Methods

### Initial System Preparation

The X-ray crystal structure of ROS1 complexed with crizotinib (PDB code 3ZBF [24], resolution 2.2 Å) was used as the initial structure for the MD simulations. The drug was optimized at HF 6-31G* level of theory using Gaussian 09 program [36], and the electrostatic potentials were calculated at the same method based on the optimized structure. The atomic partial charges were obtained by using the restrained electrostatic potential technique [37] (RESP) in Ambertools 1.5 [38].The missing residues, G1954-F1956, which are located at the P-loop region of ROS1 and were renumbered as Gly21, Ala22, and Phe23 in this study, were built with the *loop* module in SYBYL-X1.2 simulation package. Although the P-loop region was involved in the imperfect crystallization, the missing residues seemed contribute little to the binding of crizotinib with the energetic contribution ∼0 kcal/mol of the three residues as shown in Figure S3A and S3B. The residue G2032 was mutated into R2032 by using the *biopolymer* module, and followed by structural adjustment in SYBYL-X1.2. For convenience, all the residue indexes were renumbered from 1 to 285. The protonation states of residues, such as histidines and cysteines, were determined using PROPKA (version 3.1) [39]. The Amber ff99SB force field [40] and General AMBER Force Field (GAFF) [41] were used for the protein and ligand, respectively. For the unbound-state systems, crizotinib was directly removed from the crystal structures. 3 Na^+^ and 2 Na^+^ were added to neutralize the wild-type (WT) and mutated systems, respectively. Cubic TIP3P [42] water boxes were added to the systems with 10 Å extended from any solute atoms.

### Conventional Molecular Dynamics Simulations

All the simulations were performed with NAMD version 2.8 in conjunction with PLUMED 1.3 [43], [44]. A 10 Å cutoff was used for the short range electrostatic and van der Waals interactions, and the particle mesh Ewald (PME) [45] algorithm was used to handle the long-range electrostatic interactions. All covalent bonds involving hydrogen atoms were constrained with the SHAKE algorithm [46]. Prior to MD simulation, four steps of minimization were preformed to the systems. At the first stage, only hydrogen atoms were able to move freely (2500 steps). Afterward, the heavy atoms of solvent and ions were free as well (2500 steps). Thirdly, heavy atoms in side chain and backbone of protein within 5 Å of the mutation were relaxed (5000 steps). At last, all the atoms were optimized for 20,000 steps. In the MD simulation stage, the time step was set to 2 fs. The systems were gradually heated from 0 K to 310 K in 1 ns with a restrain of 5 kcal/mol·Å^2^ to the heavy atoms in the backbone in an NVT ensemble. Afterwards, the systems were relaxed for 0.2 ns with the restrain gradually decreased from 5 to 0 kcal/mol·Å^2^ in an NPT (*P* = 1 atm and *T* = 310 K) ensemble. The Poisson Piston algorithm was used to control the pressure [47]. Finally, 30 ns production runs were performed with the collective interval of 5 ps (200 frame/ns), and a total of 6000 frames were collected for the conformational space analysis.

### Metadynamics Simulations

Metadynamics simulation has been widely used in enhanced sampling simulations [48], [49], which was effective in describing free energy surface (FES) in terms of ligand-receptor binding process [50]–[54], protein conformational transition or activation [55]–[60], and protein-protein interaction [61], [62]. Moreover, numerous on-the-fly metadynamics-based techniques have been developed in recent years, such as well-tempered metadynamics [63], reconnaissance metadynamics [64], funnel metadynamics [65], parallel tempering metadynamics [56], and bias-exchange metadynamics [66], which all significantly accelerated the sampling and convergence rates compared with the standard metadynamics. Taken well-tempered metadynamics as an example, it adds a history-dependent Gaussian repulsive potential on the selected collective variables (CVs) as shown in equation (1):
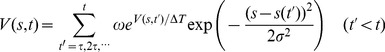
(1)where *V*(*s*, *t*) is the history-dependent biasing potential, *t*′ denotes the deposition time. At each time interval *τ*, a Gaussian potential, with the height of 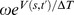, will be added on the concurrent position *s*(*t*′) of the biased molecule. Here, Δ*T* was set to 3100 K corresponding to a *bias-factor* of 10 in well-tempered algorithm. Different from the standard metadynamics that uses an immutable hill height in the simulation, the initial hill height in well-tempered metadynamics (*ω*) is scaled by the exponential of *V*(*s*, *t*′)/Δ*T* to accelerate the convergence.

Therefore, by using the advanced metadynamics techniques, namely, well-tempered metadynamics and funnel metadynamics, we explored the free energy surfaces of the crizotinib unbinding from WT and G2032R-mutated ROS1 tyrosine kinase, which were designed to move against the ligand-receptor distance and P-loop conformational change (open or close) as described in the previous studies [50], [67]. The averaged structures (derived from the equilibrium trajectories) of WT and G2032R-mutated crizotinib-ROS1 complexes were used as the initial structures for the metadynamics simulations. Prior to the metadynamics simulations, the complexes were immersed in a rectangular water box with the largest pocket direction rotated to X-axis (30 Å out of the solutes in X-axis), which was detected by Caver 2.0 [68], as we did previously [67], [69]. Then, the systems were minimized with a large restrain (100 kcal/mol·Å^2^) on the heavy atoms of the complexes. Afterward, the systems were equilibrated for 1 ns with the heavy atoms constrained as well. The final structures were submitted to the metadynamics simulations. In the well-tempered metadynamics, the heavy atoms out of 15 Å of crizotinib in proteins were constrained with 5 kcal/mol·Å^2^ to prevent drifting issues [70]. The initial hill height (*ω*) was set to 1, and the deposition rate of the added biasing potential was set to 1 kcal/mol·ps with the *bias-factor* parameter of 10 at 310 K of the simulation temperature. Two CVs were used for the construction of the free energy surface. The first CV was the distance between center-of-mass (CoM) of heavy atoms in crizotinib and center-of-mass of the binding site (heavy atoms within 5 Å of crizotinib in ROS1). The width of Gaussian hill (σ) was set to 0.4 Å, and 400 bins were divided from the range of 0 to 24 Å. The second CV was known as the similar path (S-Path) of the P-loop region of the ROS1 tyrosine kinase, which corresponds to the conformational change of the P-loop region from the bound-state conformation to the unbound-state conformation based on the measurement of Mean Square Deviation (MSD) [71]. The conformations of the bound-state and free-state P-loop were derived from the equilibrated conventional MD trajectories by measuring the dihedral angle of Cα in Glu25, Ser20, Ala22 (which are located in the P-loop region), and Arg150 (which is located in the active site with stable conformation). A total of 7 frames were used for the S-Path calculation with the MSD interval of 1 Å^2^ on average ranged from 0 to 6. The width of the Gaussian hill for the second CV was set to 0.05 Å^2^ and 350 bins were collected for the construction of FES.

Due to the hardness of convergence of the FES, funnel-based metadynamics was employed in conjunction with the well-tempered algorithm to accelerate the convergence, which adds a harmonic restrain wall around the CVs [65]. Here, a cylinder-shaped restrain funnel was constructed along the first CV (distance between CoMs of the receptor and ligand) to prevent the drug absorbing on the unrelated region of the target. The radius of the cylinder was set to 15 Å to provide enough space for the rotation of the biased molecule (∼10 Å in length of crizotinib). A restrained energy (with the elastic constant of 100 kcal/mol·Å^2^) will be added to the Hamiltonian of the biased molecule if it goes out of the cylindrical funnel for the purpose of forcing the biased molecule back to the reaction associated sampling space. Therefore, the method significantly enhances the sampling in the associated reaction-space and made the convergence of the FES rapidly. Due to the use of cylindrical restrains in the metadynamics simulations, the absolute binding free energy needs to be adjusted according to Equation (2), and the detailed descriptions could be found in reference [65].
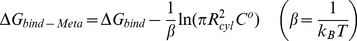
(2)where Δ*G*_bind-Meta_ represents the absolute binding free energy calculated based on metadynamics simulation, and Δ*G*_bind_ is the PMF depth corresponding to crizotinib unbounded from binding site to bulk, and 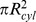 is the surface of the cylinder used in the restrains. *k_B_* is Boltzmann constant, and *C*° is the standard concentration corresponding to 1/1661 Å^3^.

To construct one-dimensional free energy profiles from two-dimensional free energy surfaces, bins with lowest energy among the S-path MSD (Y-axis in Figure 3D and 4D) were connected along the CoM distance between crizotinib and the active pocket of ROS1 tyrosine kinase (X-axis in Figure 3D and 4D).

### Umbrella Sampling (US), Adaptive Biasing Force (ABF), and Absolute Binding Free Energy Calculation

Among the enhanced sampling methodologies, umbrella sampling may be the most classic, widely used, and easily accepted method [72]–[81], which adds biasing potentials along a given reaction coordinate (RC) to drive the system from one thermodynamic state to another [82]. In detail, the RCs are usually divided into several parts, named windows, and to make things easy, harmonic potentials are often added in each window for biased sampling as shown in Equation (3).
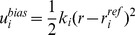
(3)where *k_i_* is the elastic constant in window *i*, and 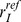 denotes the reference state of window *i*, which could be a reference conformation (RMSD), angle (α, β, γ, θ, Θ), or position (r) as shown below. After the enhanced sampling simulation, the distribution of biased samples could be unbiased to obtain the free energy change in each window. Finally, the calculated free energy in each window could be integrated by reweighted method, such as weighted histogram analysis method (WHAM) [83], [84], to give a total free energy change along the reaction coordinate.

Compared with the probability based method, such as umbrella sampling, another kind of widely used enhanced sampling approach, adaptive biasing force (ABF) [85]–[94], is the interaction-based method, which adds biasing force on the investigated molecule (or fragment) for the purpose of canceling the local barrier acted on the molecule (or fragment) [95], [96]. Therefore, as a result, all the positions of the reaction coordinate can be sampled with equal probability and the biased molecule can go with a free-diffusion-like behavior along the reaction coordinate [67], [94]. Moreover, ABF may be a more convenient method with fewer priori parameters and simulation windows needed to be tested and divided, such as it does not need a well-tested elastic constant added in each window as umbrella sampling simulation does [82], and we can use only one window to sample the orientation-associated PMFs of the biased molecule as shown below, which have a same behavior as Gumbart's result [97].

Roux and co-workers have developed a well-characterized and well-tested absolute binding free energy calculation scheme by using various restrains, including conformational, rotational, and translational restrains, to the investigated systems, which significantly accelerated the convergence due to the constriction of the relative external degrees of freedom of the systems [30], [70], [97]–[104]. In the spirit of Woo and Roux's scheme [30], the absolute binding free energy could be obtained by calculating the reaction equilibrium constant (*K*_bind_) with restrains at the biased molecule's conformation (corresponding to 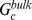 and 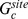), orientation (corresponding to 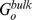, 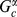, 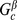, and 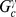), and translation (corresponding to 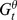, and 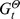), as shown in equations (4) and (5):
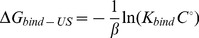
(4)

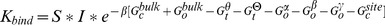
(5)where Δ*G*_bind-US_ is the absolute binding free energy calculated based on umbrella sampling simulation, and S* and I* are associated with the angular and translational restrains in bulk and separation PMF depth, respectively [30].

By using the combination of US, ABF, and Roux's absolute binding free energy calculation scheme, we accurately characterized the one-dimensional (1D) free energy profile of crizotinib separated from the binding sites of the WT and G2032R-mutated ROS1 to the bulk. Umbrella sampling was used to the conformational restrained simulations (RMSD) and separation simulations (*r*), while adaptive biasing force was employed for the angular restrained simulations, including rotational (α, β, γ) and translational (θ, Θ) associated simulations. The same well-equilibrated structures of WT and G2032R-mutated crizotinib-ROS1 complexes as those used in metadynamics simulations were employed as the initial structures for the US and ABF simulations. In the phase of conformational restrained simulations, the RMSD change of crizotinib was used as the RC for umbrella sampling (k = 0.01 kcal/mol·Å^2^). The RCs were divided into 7 and 11 windows for the bound-state and free crizotinib, respectively, with the size of each window 0.5 Å, namely, a range of 0∼3 Å and 0∼5 Å of the RMSD for the bound-state and unbound-state crizotinib. Each window was simulated for 3 ns, and the samples of the last 1.5 ns were used for the construction of the PMFs. For the angular restrained simulations, the five angles (α, β, γ, θ, and Θ) were orderly sampled with crizotinib constrained in the initial state (at the state of RMSD = 0 and a restrain of 1 kcal/mol·Å^2^ was used for the constrain of the conformation of crizotinib, and followed by the orderly restrains in the angles with 0.3 kcal/mol·Å^2^). ABF was employed for the angular restrained sampling. The bin size was set to 0.2 Å, and only one window was used for 5 ns simulations. Due to the algorithm of ABF in NAMD code, fictitious particles were used to construct an extended and generalized coordinate as proposed by Gumbart et al [97]. As shown in Figure 5, the RC of separation simulations were constructed along the vector of 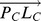, where the P_C_ point is a dummy atom placed at 5 Å away from the L_C_ point (C12 in crizotinib). 41 windows were used for the US simulation with each window 0.5 Å across the range of 5∼25 Å of the RC. The elastic constant was set to 5 kcal/mol·Å^2^ in the middle of each window to drive the drug from binding site to bulk. The initial structure in each window (except for the initial window) was dirived from the trajectory of metadynamics because metadynamics could give a most feasible unbinding pathway of the system [31]. Each window of the separation simulation was preformed with the existence of conformational and angular restrains in crizotinib. 7 ns and 3.5 ns simulations were preformed for each window involved in large (5∼13 Å) and low (13∼25 Å) barrier regions, respectively. A total of ∼200 ns simulation was preformed for each separation sampling, and the two systems both reached convergence as shown in Figure S2. The detailed simulation time can be found in Table S1. Because of the isotropy of the bulk, the energy associated with orientation in bulk could be obtained by direct numerical integration without actual MD simulation [30].

## Supporting Information

Figure S1 **Stability of the systems in conventional MD simulation.** The RMSDs and RMSFs of bound-state and unbound-state proteins are colored in grey and orange, respectively, and the ligand RMSDs are colored in green.
(TIF)

Figure S2 **Convergence of separation PMFs.** PMFs of crizotinib separated from WT-ROS1 (A) and G2032R-ROS1 (B) were obtained from 4 ns (blue), 5 ns (green), 6 ns (red), and 7 ns (cyan) extended umbrella sampling for each window (0.5 Å/window).
(TIF)

Figure S3 **Energetic contribution of important residues to the binding of crizotinib.** Energetic spectrums (enthalpy) were decomposed into drug-residue pairs for (A) wild-type ROS1, and (B) G2032R mutated ROS1, and their corresponding structural descriptions are shown in panel D and panel E, where the P-loop region and important residues on it (Leu18 and Val26) are illustrated in orange cartoon model and stick model, respectively. The mutated residue and crizotinib are modeled in green stick model (Gly99 in panel D and Arg99 in panel E) and pink stick model, respectively. The energetic difference between G2032R-ROS1 and WT-ROS1 are shown in panel C (ΔΔ*G* = Δ*G*_G2032R_−Δ*G*_WT_, a positive Δ*G* indicates a weaker binding affinity in the mutated protein, and a negative Δ*G* indicates a stronger binding affinity), where the residue Leu18 (on the P-loop region) contributes the most to the attenuated binding of crizotinib to G2032R mutated ROS1, indicating that the P-loop conformation governs the binding of crizotinib. The residue decomposition analysis was carried out by using MM/GBSA methodology, and the detailed method can be found in ref. [67].
(TIF)

Table S1 **Detailed simulation information in this study.**
(DOCX)
