# Supplementary figures and images for: P-loop Conformation Governed Crizotinib Resistance in G2032R-Mutated ROS1 Tyrosine Kinase: Clues from Free Energy Landscape
Source: PLoS Comput Biol. 2014 Jul 17;10(7):e1003729. doi: 10.1371/journal.pcbi.1003729 (PMC4102447; doi:10.1371/journal.pcbi.1003729)

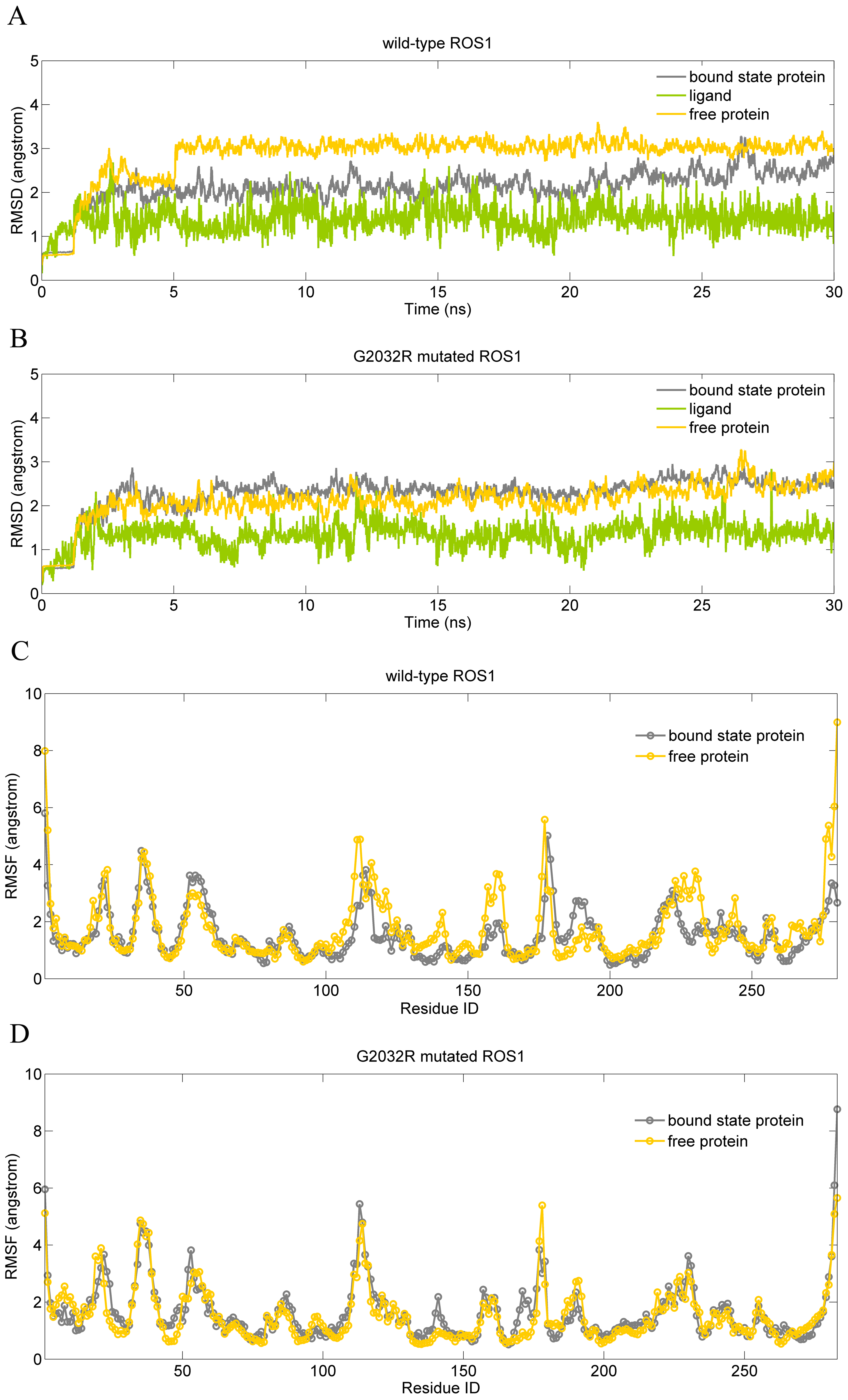

Supplement: Figure S1 — Stability of the systems in conventional MD simulation. The RMSDs and RMSFs of bound-state and unbound-state proteins are colored in grey and orange, respectively, and the ligand RMSDs are colored in green. (TIF) [file pcbi.1003729.s001.tif]

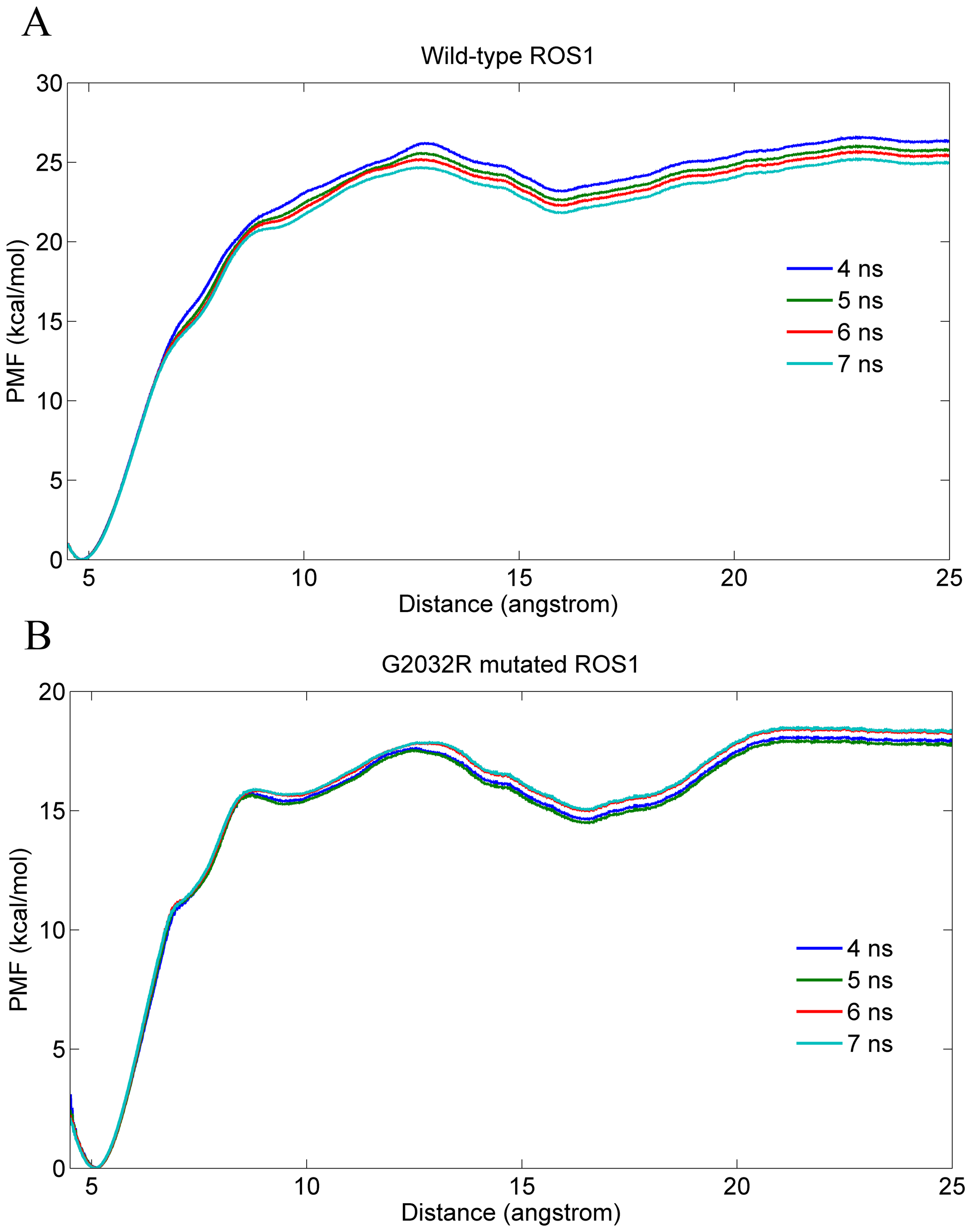

Supplement: Figure S2 — Convergence of separation PMFs. PMFs of crizotinib separated from WT-ROS1 (A) and G2032R-ROS1 (B) were obtained from 4 ns (blue), 5 ns (green), 6 ns (red), and 7 ns (cyan) extended umbrella sampling for each window (0.5 Å/window). (TIF) [file pcbi.1003729.s002.tif]

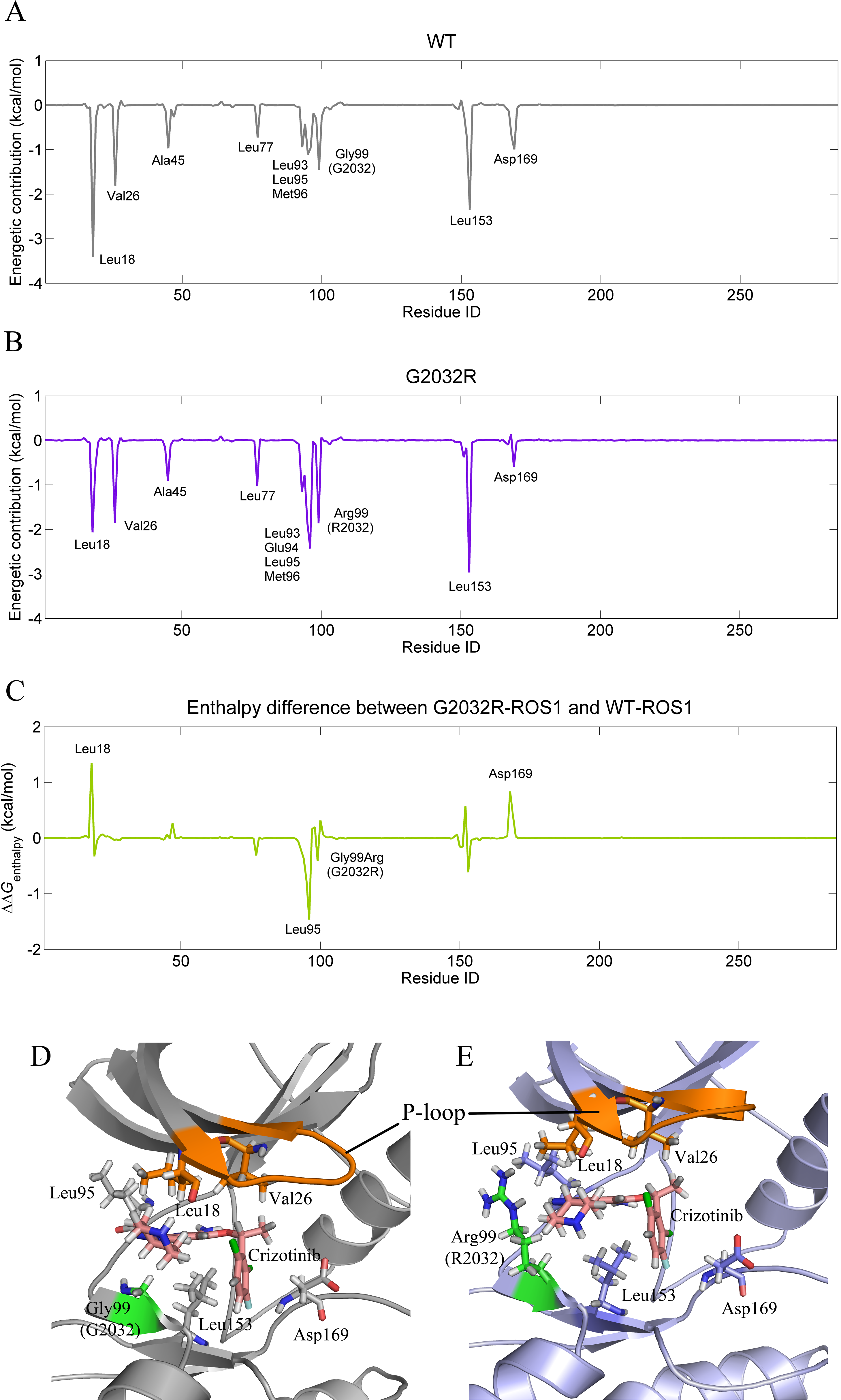

Supplement: Figure S3 — Energetic contribution of important residues to the binding of crizotinib. Energetic spectrums (enthalpy) were decomposed into drug-residue pairs for (A) wild-type ROS1, and (B) G2032R mutated ROS1, and their corresponding structural descriptions are shown in panel D and panel E, where the P-loop region and important residues on it (Leu18 and Val26) are illustrated in orange cartoon model and stick model, respectively. The mutated residue and crizotinib are modeled in green stick model (Gly99 in panel D and Arg99 in panel E) and pink stick model, respectively. The energetic difference between G2032R-ROS1 and WT-ROS1 are shown in panel C (ΔΔG = ΔG G2032R−ΔG WT, a positive ΔG indicates a weaker binding affinity in the mutated protein, and a negative ΔG indicates a stronger binding affinity), where the residue Leu18 (on the P-loop region) contributes the most to the attenuated binding of crizotinib to G2032R mutated ROS1, indicating that the P-loop conformation governs the binding of crizotinib. The residue decomposition analysis was carried out by using MM/GBSA methodology, and the detailed method can be found in ref. [67]. (TIF) [file pcbi.1003729.s003.tif]
