# Supplementary material for: P-loop Conformation Governed Crizotinib Resistance in G2032R-Mutated ROS1 Tyrosine Kinase: Clues from Free Energy Landscape
Source: PLoS Comput Biol. 2014 Jul 17;10(7):e1003729. doi: 10.1371/journal.pcbi.1003729 (PMC4102447; doi:10.1371/journal.pcbi.1003729)
Supplement: Table S1 — Detailed simulation information in this study. (DOCX) [file pcbi.1003729.s004.docx]

**Table S1.** Detailed simulation information in this study**.**

| **Simulation phase** | **Simulation time** |
| --- | --- |
| Conventional MD | 30 ns × 2 |
| Funnel based well-tempered metadynamics | 100 ns × 2 |
| RMSD restrain in binding site | 21 ns × 2 |
| RMSD restrain in bulk | 33 ns × 2 |
| α, β, γ, θ, and Θ restrained MD | 5 × 5 ns × 2 |
| Separation simulation | 196 ns × 2 |
| Total simulation time | 810 ns |
